# Contextual determinants of mass drug administration performance: Modelling fourteen years of lymphatic filariasis treatments in West Africa

**DOI:** 10.1371/journal.pntd.0011146

**Published:** 2023-02-24

**Authors:** Brian B. Fuller, Vance Harris, Caleb Parker, Andres Martinez, Emily Toubali, Blandine Clarisse Ebene, Kofi Asemanyi-Mensah, Massitan Dembele, Adamou Bacthiri Salissou, Cathérine Kabré, Aboulaye Meite, Ndeye Mbacke Kane, Ibrahim Kargbo-Labour, Wilfrid Batcho, Aissatou Diaby, Violetta Yevstigneyeva, Diana Maria Stukel

**Affiliations:** 1 Helen Keller International, Washington, District of Columbia, United States of America; 2 FHI 360, Denver, Colorado, United States of America; 3 FHI 360, Durham, North Carolina, United States of America; 4 Division of Neglected Tropical Diseases, Office of Infectious Diseases, Bureau for Global Health, USAID, Washington, District of Columbia, United States of America; 5 National Programme for Onchocerciasis and Lymphatic Filariasis Control, Ministry of Public Health, Yaoundé, Cameroon; 6 Neglected Tropical Diseases Programme, Disease Control and Prevention Department, Ghana Health Service, Public Health Division, Accra, Ghana; 7 National Programme for the Elimination of LF, Ministry of Health, Bamako, Mali; 8 Programme Onchocercose et Filariose Lymphatique, Ministry of Health, Niamey, Niger; 9 Programme national de lutte contre les maladies tropicales négligées, Ministry of Health, Ouagadougou, Burkina Faso; 10 Programme national de lutte contre les maladies tropicales négligées à chimiothérapie préventive, Ministry of Health, Abidjan, Côte d’Ivoire; 11 National Neglected Tropical Diseases Control Program, Ministry of Health, Dakar, Senegal; 12 Neglected Tropical Disease Programme, Ministry of Health and Sanitation, Freetown, Sierra Leone; 13 Programme National de Lutte contre les Maladies Transmissibles, Ministry of Health, Cotonou, Benin; 14 National Neglected Tropical Diseases Control Program, Ministry of Health, Conakry, Guinea; 15 Act to End NTDs | West, FHI 360, Washington, District of Columbia, United States of America; Erasmus MC, NETHERLANDS

## Abstract

**Background:**

Effective mass drug administration (MDA) is the cornerstone in the elimination of lymphatic filariasis (LF) and a critical component in combatting all neglected tropical diseases for which preventative chemotherapy is recommended (PC-NTDs). Despite its importance, MDA coverage, however defined, is rarely investigated systematically across time and geography.

Most commonly, investigations into coverage react to unsatisfactory outcomes and tend to focus on a single year and health district. Such investigations omit more macro-level influences including sociological, environmental, and programmatic factors.

The USAID NTD database contains measures of performance from thousands of district-level LF MDA campaigns across 14 years and 10 West African countries. Specifically, performance was measured as an MDA’s epidemiological coverage, calculated as persons treated divided by persons at risk.

This analysis aims to explain MDA coverage across time and geography in West Africa using sociological, environmental, and programmatic factors.

**Methodology:**

The analysis links epidemiological coverage data from 3,880 LF MDAs with contextual, non-NTD data via location (each MDA was specific to a health district) and time (MDA month, year). Contextual data included rainfall, temperature, violence or social unrest, COVID-19, the 2014 Ebola outbreak, road access/isolation, population density, observance of Ramadan, and the number of previously completed MDAs.

**Principal findings:**

We fit a hierarchical linear regression model with coverage as the dependent variable and performed sensitivity analyses to confirm the selection of the explanatory factors.

Above average rainfall, COVID-19, Ebola, violence and social unrest were all significantly associated with lower coverage. Years of prior experience in a district and above average temperature were significantly associated with higher coverage.

**Conclusions/Significance:**

These generalized and context-focused findings supplement current literature on coverage dynamics and MDA performance. Findings may be used to quantify typically anecdotal considerations in MDA planning. The model and methodology are offered as a tool for further investigation.

## Author summary

Elimination of lymphatic filariasis, and of certain other neglected tropical diseases, depends critically on the successful treatment of the population through a series of typically annual mass drug administration (MDA) campaigns. When an MDA falls short of expectations in terms of population treated, organizers can learn and adapt the next round of MDA to improve performance. Because this learning is often based on anecdotal evidence or surveys specific to a narrow geography, there is an opportunity in the literature to augment our understanding of what impacts MDA performance, particularly in terms of more contextual factors and trends or patterns that reach across multiple years and regions.

In this analysis we linked a performance measure from 3,880 MDA campaigns across 10 West African countries to contextual factors such as rainfall, temperature, violence or social unrest, COVID-19, the 2014 Ebola outbreak, road access/isolation, population density, observance of Ramadan, and the number of previously completed MDAs. We hope the findings themselves will better equip MDA organizers in decision-making and offer this mixed-level model as an approach to better understanding coverage dynamics in West Africa and elsewhere.

## Introduction

Lymphatic filariasis (LF) is a debilitating and disfiguring neglected tropical disease (NTD) with an estimated 863 million people in need of treatment across 72 endemic countries as of 2020 [1]. To stop the spread of LF infection and alleviate suffering, the World Health Organization (WHO) created the Global Programme to Eliminate Lymphatic Filariasis (GPELF) in 2000. GPELF guidance requires multiple rounds of mass drug administration (MDA) of ivermectin with diethylcarbamazine (DEC) or albendazole [2]. These MDA rounds should achieve “effective” coverage of an endemic area by, during each round, treating at least 65% of the population at risk, typically considered the full population of the endemic area [1,3]. This coverage rate, known as epidemiological coverage, typically includes all residents of an endemic area in the denominator. Using annual MDA and diagnostics to subsequently validate interrupted transmission, twenty-five countries globally have reached the criteria to stop MDA, among which seventeen have officially validated the elimination of LF as a public health problem [1].

With such importance on coverage, analyses of LF MDA coverage—particularly diagnosing poor coverage—are numerous and geographically diverse, including South Asia [4–6], Southeast Asia [7], Caribbean [8,9], Africa [10–12], as well as multi-country reviews [13,14].

Insights into the performance of MDA campaigns need not be limited to those treating LF; other preventive chemotherapy NTDs (PC-NTDs) are treated in similar MDA campaigns where hundreds of community drug distributors (CDDs) are trained and mobilized to administer treatment to all or nearly all the population. Given this logistical similarity of LF campaigns to other NTD campaigns, evidence and lessons learned from trachoma and onchocerciasis MDAs can be directly applied to those of LF and vice versa. This evidence includes post-MDA coverage studies of trachoma treatment coverage [15] and onchocerciasis treatment coverage [16,17]. Analyses of MDA coverage for schistosomiasis [18,19] and soil-transmitted helminths [20] are also instructive, though treatments are often, but not always, administered via schools in contrast to the community-wide distribution typical of LF and trachoma. Like the majority of analyses into the dynamics behind LF MDA coverage, these NTD MDA coverage analyses often make use of the post-MDA coverage evaluation survey (CES), a population-based survey tool designed to provide precise estimates of MDA coverage and evaluate programmatic performance [21].

Studies based on the CES tool or those like it are typically limited to analyzing district-level coverage, with said districts chosen purposively. With the general public as respondents, such surveys naturally focus on the publicly understood aspects of the campaign (e.g., social mobilization, prior knowledge of the disease itself) or demographic determinants of participation (e.g., age, gender, educational attainment, ethnic/linguistic group membership, occupation). Both the scale (local district) and content (MDA participant-centered) of CES based studies reveal an opportunity to supplement current research with more generalizable, context-oriented analyses.

A limited number of larger studies have generalized findings to a wider context but maintain a focus on participant-level attributes, particularly in sub-Saharan Africa where the majority of endemic countries are found [1]. These can be meta-analyses of CES-based papers [13] or comparisons of the MDA’s own reported coverage against individual-level data such as gender [14]. A small subset of these larger studies has incorporated more macro-level explanatory factors of coverage such as delivery platform [22], the number of drug distributors and their incentives [23], or provision of other health services like vitamin A [24].

Unlike the metrics of “coverage” associated with clinic-based health services, the coverage of NTD MDA campaigns is reliant on the logistics of travel and local delivery and is therefore tied to contextual, location-specific factors such as weather, road conditions, political unrest as well as an organizer’s experience implementing in the area. CES studies frequently identify failures in drug delivery or poor drug access [5–7,9,10,12] (as opposed to refusals or other barriers to participation) as a key driver for low coverage but are, by design, limited in their ability to identify the underlying causes behind a failure to deliver treatment.

The goal of this study is to better understand the macro-level environmental, social, and programmatic factors influencing LF MDA campaigns, and in so doing, equip campaign organizers within and outside the NTD world with an understanding of ideal and detrimental conditions for future MDA campaigns.

## Methods

This study considered LF treatment and population data provided to USAID implementing partners by the national NTD programs of Benin, Burkina Faso, Cameroon, Guinea, Ghana, Côte d’Ivoire, Mali, Niger, Senegal, and Sierra Leone. Coverage data for 3,880 MDA campaigns in 647 districts between 2007–2020 were provided at the district level, the unit of analysis for this study. Unless otherwise specified, an MDA refers to a treatment campaign belonging to a specific month, year, and district.

### Hierarchical linear model

This analysis used a hierarchical linear model (HLM), also known as a multi-level model, to model the epidemiological coverage rate of 3,880 MDA events in 647 districts belonging to 10 countries, as a function of programmatic, sociological, and environmental explanatory variables. The variables used are described in Table 1. The HLM allows us to properly account for the nested nature of the data (i.e., MDA events nested within districts nested within countries) and to test the hypotheses that a country and district’s inherent characteristics influence coverage rates independent of event specific factors. In contrast to analyses of variance that neglect within-group dependencies, HLMs differentiate the within- and between-group variances, resulting in more accurate estimates of the associations between the outcome variable and the explanatory variables [25] operating at the MDA event, district, and country levels.

**Table 1:** Summary of variables included in models.

Variable	Description	Mean	Min	Max	SD	Source
Coverage	Epidemiological coverage rate: % of population treated in a given MDA event	72.6%	24.5%	121.0%	14.0%	USAID NTD Database
Month of MDA	Eleven month-specific variables indicating if the MDA event began in that month (1 = yes; 0 = no. May kept as reference)	N/A	N/A	N/A	N/A	USAID NTD Database
Increased rainfall	Actual rainfall minus average rainfall (mm per day)	-0.79	-12.06	11.01	2.04	NOAA PSL
Increased temperature	Actual temperature minus average temperature (Celsius)	0.62	-7.77	7.32	1.57	NOAA PSL
Round of MDA	An MDA event’s position in a district’s sequence history of MDA events (i.e., first, second, third, etc.). Included in the model as round-specific dummies, keeping the 1^st^ round as reference.	3.94	1	12	2.31	USAID NTD Database
MDA conducted after hiatus	A given LF MDA followed > 1 year of no LF MDA (1 = yes; 0 = no)	9.6%	0	1	29.5%	USAID NTD Database
MDA conducted same month as previous MDA	A given LF MDA occurred in the same month as the district’s previous LF MDA (1 = yes; 0 = no)	34.8%	0	1	47.7%	USAID NTD Database
Focal MDA	LF MDA targeted a subset of district’s population (1 = yes; 0 = no)	7.8%	0	1	26.9%	USAID NTD Database
Ramadan	LF MDA began in the same month as Ramadan (select geographies) (1 = yes; 0 = no)	9.2%	0	1	28.9%	Habibur (dates of observance).
COVID-19	LF MDA occurred during the COVID-19 pandemic (1 = yes; 0 = no)	2.6%	0	1	15.8%	CDC (dates of outbreak)
Ebola	LF MDA occurred during Ebola outbreak (1 = yes; 0 = no)	0.6%	0	1	8.0%	CDC (dates of outbreak)
Violence/Social unrest (any type)	≥ 1 occurrence of any of six types of unrest concurrent with MDA or the month prior (1 = yes; 0 = no)	6.5%	0	1	24.6%	ACLED
Battles	≥ 1 battle concurrent with MDA or the month prior (1 = yes; 0 = no)	2.2%	0	1	14.6%	ACLED
Explosions/Remote violence	≥ 1 explosions or remote violence concurrent with MDA or the month prior (1 = yes; 0 = no)	0.6%	0	1	8.0%	ACLED
Protests	≥ 1 protest concurrent with MDA or the month prior (1 = yes; 0 = no)	2.0%	0	1	14.1%	ACLED
Riots	≥ 1 riot concurrent with MDA or the month prior (1 = yes; 0 = no)	2.5%	0	1	15.5%	ACLED
Violence against civilians	≥ 1 occurrences of violence against civilians concurrent with MDA or the month prior (1 = yes; 0 = no)	1.7%	0	1	13.0%	ACLED
Looting/Property destruction	≥ 1 occurrence of looting or property damage concurrent with MDA or the month prior (1 = yes; 0 = no)	0.1%	0	1	3.6%	ACLED
Isolation	% Of a given district’s population living > 2 hours travel time from a major or minor road	12.0%	0%	100%	17.7%	OSM; HumData; Globcover
Population density	(Quintile of) District population/km2. Included in Model 1 as quintile-specific dummies, keeping the 1^st^ quintile as reference.	0.29	<0.01	19.28	1.22	USAID NTD Database; Act | West GIS

The model was formulated as:

Y_*ijk*_ = *β*_0_ + $\sum_{m=2}^{12}$ *γ*_m_(month)_mijk_ + *β*_1_(Increased rainfall)_ijk_ +*β*_2_(Increased temperature)_ijk_ + $\sum_{m=2}^{12}$ *α*_m_(round)_mijk_ + *β*_3_(MDA conducted after hiatus)_ijk_ + *β*_4_ (MDA conducted same month as previous MDA)_ijk_ + *β*_5_(focal)_ijk_ + *β*_6_(ramadan)_ijk_ + *β*_7_(covid-19)_ijk_ + *β*_8_(ebola)_ijk_ + *β*_9_(Violence/Social unrest (any type))_ijk_ + *π*_1_(isolation)_ijk_ + $\sum_{m=2}^{5}$ *δ*_m_(population density group)_mijk_ + *e*_*ijk*_ + *r*_*jk*_ + *u*_*k*_

Where Y_*ijk*_ is the epidemiological coverage rate from an MDA event *i* in district *j* in country *k*. Time i.e., calendar year was not formally included in the model due to collinearity with round (districts typically conduct one round per calendar year, with some exceptions) and the sequential progression of rounds was deemed more explanatory of coverage than calendar year. The terms $\sum_{m=2}^{12}$ *γ*_m_(month)_mijk_, $\sum_{m=2}^{12}$ *α*_m_(round)_mijk_, and $\sum_{m=2}^{5}$ *δ*_m_(population density group)_mijk_ represent series of related dummy variables (e.g., round 2, round 3, round 4…; January, February, March…) with each dummy having a distinct coefficient. Note that “round” refers to when a given MDA campaign event fell in the overall history of a district’s LF MDAs (i.e. 1^st^, 2^nd^, 3^rd^, etc..) and did not refer to its order within a single year.

Two distinct models were fitted to the data. The first model included all the variables in Table 1 except for isolation and the six dummy variables related to violence/unrest (i.e., battles, explosions/remote violence, protests, riots, violence against civilians, and looting/property destruction). The model also included dummy variables to represent the calendar months (one for each month except for May, the reference) and the round (one for each round, except the first, which is the reference). The second model differed in two ways. First, the six dummy variables related to violence/unrest were included in lieu of the single variable representing violence/unrest of any type. Second, we used the variable isolation in lieu of the series of dummy variables representing population density quantiles used in Model 1. These two specifications allow results and discussion of two related but distinct concepts, i.e., population density and isolation, without collinearity within a single model. Model 2 also expands on the Model 1 estimate of any unrest with specific types of unrest and their respective estimates. The analysis was conducted using Stata 15 SE [26].

### Programmatic and NTD data

#### Treatment and population

The LF MDA campaign functions both as a treatment delivery and data collection platform. For the LF treatment campaign considered in this study, community drug distributors (CDDs) responsible for administering the correct dose of albendazole and ivermectin also record the number of persons treated on a paper tally sheet. After, or in some cases during the MDA, this number is aggregated up through intermediate administrative levels (e.g., supervisory area, chiefdom, health area) to the health district, hereafter synonymous with implementation unit (IU). The total number of LF treatments in each treated district is reported to the national NTD program.

Population data were provided by the national NTD programs for each district conducting LF MDA. The data were provided each year, accounting for population growth, and were typically based on a national census. In certain years, a minority of the NTD programs included opted to use NTD-specific population estimates for (e.g., a pre-MDA community census conducted by the CDDs themselves). This was done in cases of population movement or where the national census was considered unreliable or too old to be accurate.

Treatment and population data were considered exactly as reported to implementing partners following the MDA and national validation. All treatment and population data were recorded for the district level along with the month and calendar year of the MDA start. All treatment, population, and calendar data were sourced directly from the USAID NTD database for countries currently participating in USAID’s Act to End NTDs | West program. District geographic boundary data (i.e. shapefiles) was also shared by the ten ministries of health to implementing partners during the course of implementation.

#### MDA coverage rates

This study calculated an epidemiological coverage rate for each of the district-specific MDA campaigns as the number of persons treated in a district’s MDA campaign divided by that district’s population. The full district population was considered at risk for LF as ascertained by LF mapping prior to the recorded MDAs. The values of persons treated and population were not altered from those provided by national NTD programs. The epidemiological coverage rate was the dependent variable in both models.

Coverage rates that fell outside three standard deviations from their mean were considered outliers, and were capped at that value (i.e., mean plus/minus three standard deviations). These capped values were 24.6% and 121.0%. (Note: Factors such as population movement make >100% coverage a valid result worth inclusion in this study.) Around 1.5% of the rates were capped in this way. A sensitivity analysis was conducted for capping at 2 standard deviations (SD), 2.5 SD, and no capping to ensure results were robust to data capping decisions. Where records showed an MDA was focal, (defined for this study as targeting less than 75% of the district population) a dummy variable was used to control for this in terms of coverage.

#### Programmatic aspects of MDA

Variables for treatment round, treatment hiatus, and consistency in MDA scheduling were derived from the month, year, and district data associated with each MDA event. A value for MDA round ranging from 1–12 was assigned to each MDA indicating it was the *n*th LF MDA conducted in the district. A binary treatment hiatus variable was created to note which MDAs, being typically annual for LF, occurred following a year or more pause in treatment (e.g., insecurity, labor strikes, or following surveys that signaled a need for renewed MDA). A third variable attempted to capture consistency in MDA scheduling; the binary variable is true if an MDA is implemented in the same month as the previous round, plus or minus one month. Finally, the calendar months themselves were introduced to the model as control variables (12, one month omitted) to separate unobservable factors associated with a given month from the estimates of the factors of interest.

### Sociological data

#### Political violence and protest

Data on violence and unrest were derived from the Armed Conflict Location & Event Data Project (ACLED), which is a disaggregated data collection, analysis, and crisis mapping project. The nonprofit group provides regularly updated data on political violence and protest globally and for the ten countries analyzed in this study since 1997 [27]. The ACLED dataset categorizes 22,868 events falling within the scope of this study into six categories (i.e., battles, explosions/remote violence, protests, riots, strategic developments, and violence against civilians) and 24 subcategories [28]. As events in the strategic developments category varied widely and were more conceptually abstract (e.g., “change to group/activity”), the analysis considered only event subtypes with a clear and plausible connection to MDA performance (e.g., looting/property destruction). ACLED data were prepared for this analysis by linking each event temporally (month and year) and geographically (district) to each of the MDA campaigns included in the dataset. Events were associated with a given MDA if they coincided with the month of or preceding MDA implementation. This captured the influence of such events on pre-MDA social mobilization as well as implementation itself.

#### Population density

To assess how rural and urban settings affected MDA coverage, the study calculated population density by dividing the district population reported by the national NTD programs by the district area in square kilometers. Similar to all other variables used, geographic shapefiles reflected each country’s health district boundaries as of June 1, 2021. These 2021 geographical boundaries were imposed retroactively on districts that merged or split during the years of this study (2007–2020), allowing continuity of each district backwards in time. This data management step was never taken during an MDA campaign. Since both extremes of population density–the most rural and most urban—are recognized as presenting distinct challenges to an MDA, the relationship between population density and coverage was not expected to be linear or logarithmic. Thus, districts and their population densities were classified into one of five quantiles ranging from most rural (quantile 1) to most urban (quantile 5) allowing each quantile to have its own association with coverage. Specifically these quantiles were defined as .1–24.7 population per km^2^ (quantile 1, most rural 20% of values), 24.7–53.4 (quantile 2), 53.4–96.2 (quantile 3), 96.3–200.7 (quantile 4), and 200.8–19,283.5 (quantile 5, most urban 20% of values).

#### Isolation/Road access

The proportion of each district’s population that lived beyond a two-hour travel-time threshold was calculated to generate a proxy metric for community isolation. Travel-time was selected over distance as our metric of isolation as it more directly impacts a community’s accessibility to the surrounding region. An assumption was made that areas not directly connected to the road-network would be traveled on foot, therefore would be more influenced by the surrounding land cover. Using the district road networks [29], population data [30], and land cover/land use data [31], a cost distance raster, used to calculate movement over continuous space, in which the cost of moving through any location is variable, was created to quantify the total travel times from any single point in the district to the nearest major or minor road [32,33]. A binary raster layer was then created to delineate areas with each district based on the two-hour threshold. The percentage of the population residing outside the threshold (> 2 hours travel from a major or minor road) was then calculated, with higher values indicating more of the district’s residents were hard to reach. The two-hour travel-time threshold was selected after a sensitivity analysis was performed on different travel-time intervals.

#### Ramadan

A binary variable was created to assess the influence of the holy month of Ramadan, where observed, on MDA coverage. This variable was true if a) the MDA took place in a predominantly Muslim country (Niger, Senegal, Mali) or a majority Muslim region in other countries [34], and b) the start month of MDA coincided with the dates of Ramadan for that year [35]. As the exact first and last dates of MDA were not available to compare to those of Ramadan, this variable represents proximity to and/or overlap with Ramadan, i.e., occurring in the same Gregorian calendar month. The variable was designed to broadly capture both direct (e.g., a refusal to take drugs on an empty stomach) and indirect (e.g., insufficient drug stock from logistical delays due to modified business hours) effects of the observance.

#### COVID-19 and Ebola virus disease outbreaks

Two binary variables were created to denote whether an MDA occurred during the Ebola or COVID-19 outbreaks. These variables were intended to capture the influence of the outbreaks on the MDA campaign (e.g., potential changing attitudes toward interactions with strangers) and the increased logistical burden of conducting an MDA in accordance with national, WHO, and donor preventative measures [36]. For COVID-19, one hundred MDA campaigns were held between August and December 2020, each of which represented a district’s first LF MDA adapting to the new guidance on preventative measures. For Ebola, 16 district-level MDA campaigns in Sierra Leone and Guinea were conducted between March 2014 and December 2015. [37]

### Environmental data

#### Temperature and precipitation

To understand how weather could impact coverage, temperature [34] and precipitation [35] data were gathered for each month an LF MDA occurred and compared to the 30-year average for that district from July 1991 to July 2021. Temperature was measured in average degrees Celsius over the month and precipitation was measured in average daily accumulation in millimeters (mm) throughout the month. The variables in the model are the difference between a district’s 30-year average temperature or precipitation and the observed measurements. Specifically, Increased temperature was calculated as *actual* temperature (degrees Celsius) during a given MDA for a given district minus *average* temperature for said district at that time of year. Positive values indicate the MDA was conducted in hotter than normal conditions, while negative values indicate cooler than normal conditions. Increased rainfall was calculated similarly such that positive values indicate the MDA was conducted in wetter than normal conditions and vice versa. A summary of all programmatic, sociological and environmental variables used is included in Table 1 below.

### Sample description

**Fig 1:**
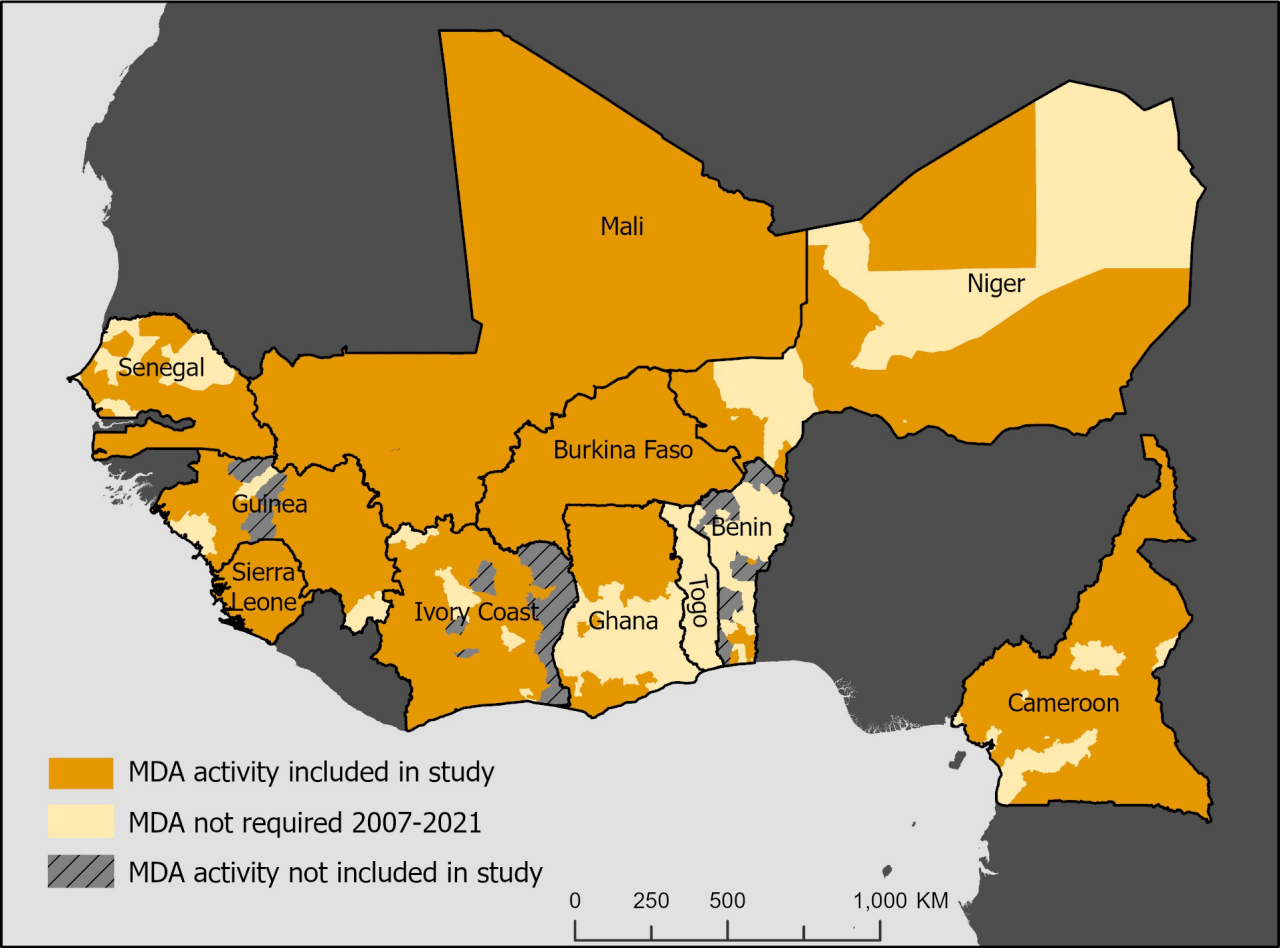
Geographical distribution of LF MDA data included in study. Created using a base layer from the Database of Global Administrative Areas (GADM) (source: https://gadm.org/download_world.html).

The scope of analysis included USAID-supported MDA campaigns in nine West African countries and Cameroon from 2007 to 2020 (Table 2). The sample included 3,880 USAID-supported MDA campaigns across 647 districts and 102 regions within the 10 countries studied (Fig 1), as defined by June 2021 geographic listings. Each district conducted between 1 and 12 MDA rounds (mean 3.94; SD 2.31). Epidemiological coverage averaged 72.6% (SD 14.0). The MDAs detailed in Table 2 are considered district-specific events. Because Table 2 data reflect redistricting (i.e., reclassifying historical data in terms of 2021 district boundaries maintained within the USAID NTD database) and the exclusion of non-USAID supported MDAs, totals may differ from country- or WHO-maintained data sources. Table 2 is presented as a research sample description and not an authoritative record of country LF MDA history. Across all years, MDA implementation favored the months of April, May, June, and July, together accounting for 61.7% of all implemented MDAs. MDAs were implemented the least frequently in the month of October (2.4% of MDAs) likely due to MoH preference and the transition of the USAID fiscal year.

**Table 2:** Selected LF MDA campaigns included in analysis, by country and year, adjusted to 2021 administrative boundaries.

Year of MDA	Benin	Burkina Faso	Cameroon	Ghana	Guinea	Côte d’Ivoire	Mali	Niger	Senegal	Sierra Leone	Total
2007	0	0	0	88	0	0	30	17	0	0	**135**
2008	0	0	0	88	0	0	41	30	0	15	**174**
2009	0	63	0	0	0	0	65	36	0	14	**178**
2010	0	70	44	196	0	0	65	40	1	16	**432**
2011	0	33	129	0	0	0	65	52	1	16	**296**
2012	0	43	136	93	0	0	0	52	1	16	**341**
2013	25	36	136	93	0	0	0	2	14	16	**322**
2014	25	41	136	93	4	0	17	50	7	0	**373**
2015	25	26	146	30	9	24	63	48	52	16	**439**
2016	25	26	136	22	16	28	73	40	35	16	**417**
2017	25	20	92	17	19	74	18	37	34	8	**344**
2018	12	4	1	15	19	76	0	9	31	6	**173**
2019	4	9	0	15	0	75	0	11	31	7	**152**
2020	4	5	0	0	19	54	0	0	22	0	**104**
**Total**	**145**	**376**	**956**	**750**	**86**	**331**	**437**	**424**	**229**	**146**	**3,880**

## Results

Table 3 shows the estimates for each parameter included in both hierarchical linear models. In Model 1 the country level variance was .0024 (SD .0011), the district level variance was .0033 (SD .0003) and the event level variance was .0126 (SD .0003). In Model 2 the country level variance was .0026 (SD .0012), the district level variance was .0033 (SD .0003) and the event level variance was .0126 (SD .0003). For point estimates appearing in both models, the text below will refer to estimates in Model 1 unless otherwise noted.

**Table 3:** Results of hierarchical linear models.

	Model 1			Model 2		
Covariate	Estimate	SE	P-value	Estimate	SE	P-value
Month of MDA						
Jan	0.0407	0.0125	0.001*	0.0384	0.0125	0.002*
Feb	0.0372	0.0136	0.006*	0.0364	0.0137	0.008*
Mar	-0.0261	0.0112	0.020*	-0.0279	0.0112	0.013*
Apr	-0.0087	0.0094	0.352	-0.0100	0.0094	0.287
Jun	-0.0068	0.0068	0.319	-0.0079	0.0068	0.244
Jul	-0.0221	0.0069	0.001*	-0.0226	0.0069	0.001*
Aug	0.0057	0.0104	0.584	0.0039	0.0104	0.708
Sep	0.0331	0.0127	0.009*	0.0303	0.0127	0.017*
Oct	0.0081	0.0145	0.577	0.0075	0.0145	0.608
Nov	0.0160	0.0144	0.267	0.0156	0.0145	0.280
Dec	-0.0257	0.0109	0.018*	-0.0266	0.0109	0.015*
Increased rainfall	-0.0027	0.0012	0.027*	-0.0028	0.0012	0.024*
Increased temperature	0.0080	0.0020	<0.001*	0.0075	0.0020	<0.001*
Round of MDA						
2	0.0349	0.0071	<0.001*	0.0347	0.0071	<0.001*
3	0.0594	0.0084	<0.001*	0.0595	0.0084	<0.001*
4	0.0519	0.0086	<0.001*	0.0524	0.0086	<0.001*
5	0.0824	0.0090	<0.001*	0.0821	0.0090	<0.001*
6	0.0826	0.0090	<0.001*	0.0831	0.0090	<0.001*
7	0.0999	0.0094	<0.001*	0.1007	0.0094	<0.001*
8	0.0871	0.0146	<0.001*	0.0891	0.0146	<0.001*
9	0.0788	0.0181	<0.001*	0.0827	0.0181	<0.001*
10	0.0927	0.0194	<0.001*	0.0938	0.0193	<0.001*
11	0.1017	0.0247	<0.001*	0.1036	0.0247	<0.001*
12	0.0969	0.0316	0.002*	0.0972	0.0316	0.002*
MDA conducted after hiatus	0.0004	0.0081	0.961	0.0025	0.0081	0.754
MDA conducted same month as previous MDA	0.0163	0.0060	0.007*	0.0162	0.0060	0.007*
Focal MDA	-0.0841	0.0084	<0.001*	-0.0842	0.0084	<0.001*
Ramadan	0.0099	0.0077	0.196	0.0108	0.0077	0.159
COVID-19	-0.0466	0.0162	0.004*	-0.0460	0.0163	0.005*
Ebola	-0.0733	0.0255	0.004*	-0.0741	0.0255	0.004*
Violence/Social unrest						
Violence/Social unrest (any type)	-0.0319	0.0085	<0.001*			
Battles				-0.0018	0.0151	0.908
Explosions/Remote violence				-0.1055	0.0270	<0.001*
Protests				-0.0219	0.0151	0.147
Riots				-0.0254	0.0137	0.064
Violence against civilians				-0.0171	0.0165	0.301
Looting/Property destruction				0.0530	0.0543	0.329
Isolation				-0.0003	0.0002	0.077
Population density (population/km^2^)						
Group 2: 25–53	0.0213	0.0085	0.012*			
Group 3: 53–96	0.0159	0.0090	0.076			
Group 4: 96–201	0.0176	0.0092	0.057			
Group 5: 201–19283	0.0061	0.0097	0.528			
Constant	0.6486	0.0192	<0.001*	0.6653	0.0191	<0.001*

* Indicates significance at the 5% level

Above average rainfall during MDA, when compared to a district’s climatological average for the same month, was significantly associated with lower coverage. For each mm/day (averaged across the month) above the 30-year average, coverage was reduced by 0.27 percentage points. Above average heat was significantly associated with increased coverage, however. For each additional degree Celsius above the 30-year average, coverage was 0.80 percentage points higher. Both results were robust with the inclusion of calendar month dummies.

**Fig 2:**
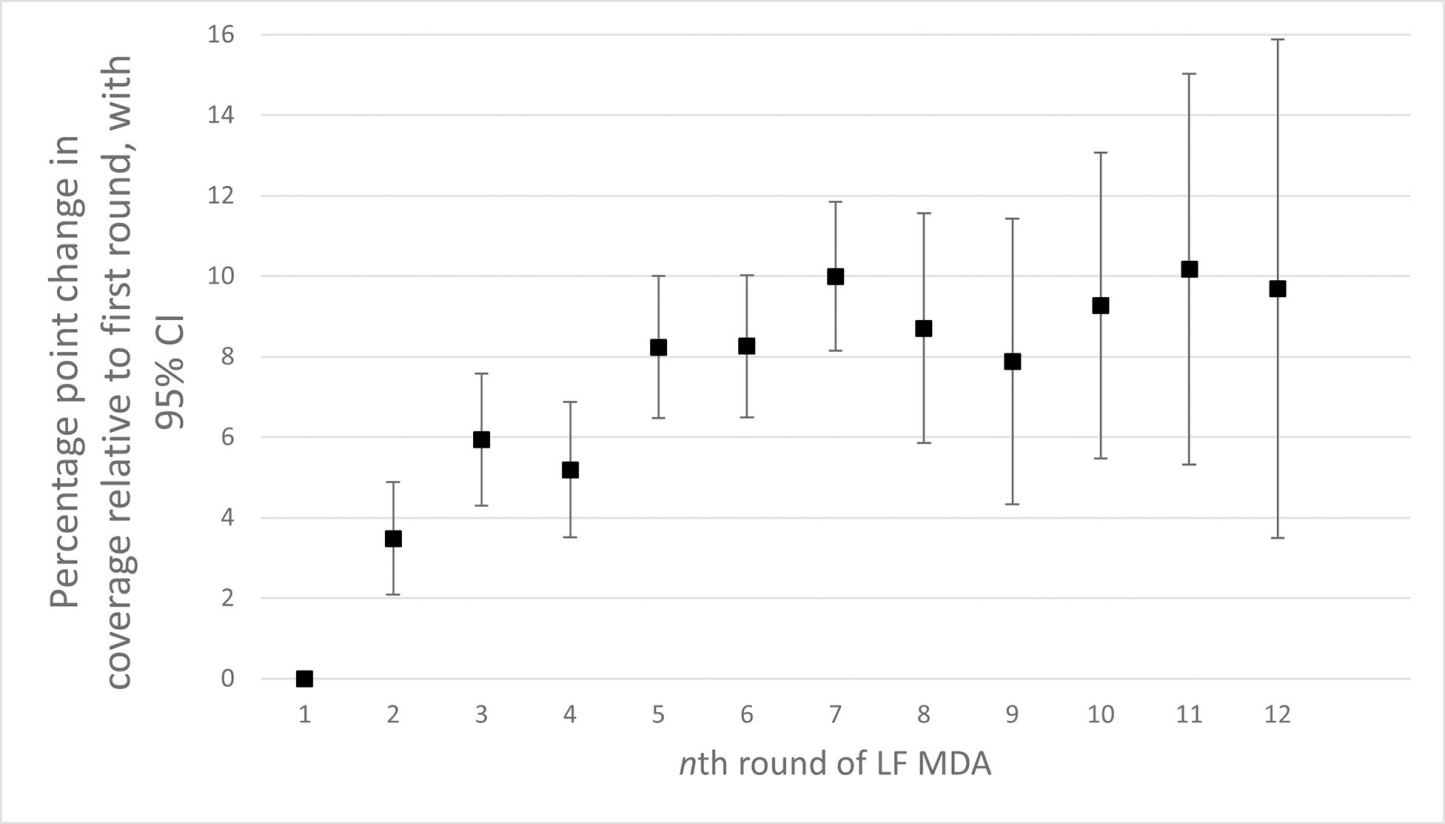
Change to coverage with each additional round of MDA.

Coverage increased with successive rounds of MDA, from a 3.5 percentage point increase for a second round compared to the first, to approximately ten percentage points in rounds 10–12 relative to round one (Fig 2). A hiatus, where more than a year of no LF MDA passed before a given MDA began, was not significantly associated with coverage. This included districts implementing after a planned hiatus (e.g., a scheduled LF pre-Transmission Assessment Survey) or unplanned hiatus (e.g., a health worker strike preventing MDA implementation). Conducting the MDA in the same calendar month as the year before was associated with 1.6 percentage points of improved coverage. Where records showed an MDA was focal, (defined for this study as targeting less than 75% of the district population), this was associated with significantly lower epidemiological coverage (8.4 percentage points, p<0.001). Note that epidemiological coverage was calculated using a district’s full population in the denominator regardless of the number of persons targeted by MDA organizers.

There was no significant effect on coverage in cases where MDA happened within the same month as Ramadan in a Muslim-majority region or country.

The MDAs that took place during the COVID-19 pandemic achieved lower coverage by 4.7 percentage points (p = .004). This data reflected MDAs in 100 districts across Burkina Faso, Guinea, Côte d’Ivoire and Senegal, all starting between August and December 2020. In each of these 100 districts, these MDAs were the first to coincide with this pandemic. MDAs that took place during the 2014–2015 Ebola virus outbreak in Guinea and Sierra Leone, 25 MDAs in all, had 7.3 percentage points (p = .004) lower coverage.

MDAs that took place the same or following month as recorded unrest or violence (n = 252) achieved significantly lower coverage (-3.2pp; p<0.001). From Model 2, explosions/remote violence was significantly associated with lower coverage (-10.5pp, p<0.001). The remaining event-type specific estimates were not significant at the 95% level. These six event types were relatively rare amongst the 3,880 MDA studied; the most frequently occurring was riots, linked to 96 MDAs.

In terms of population density, Model 1 showed lowest coverage associated with the most sparsely populated 20% of districts (<25 population per km2), omitted from the model. Group Two (25–54 population per km2) by contrast was 2.1 percentage points higher (p = 0.012). In terms of road access, Model 2 suggests less access is minimally detrimental to coverage though this parameter is narrowly insignificant at the 95% level (-.03 percentage points, p = .077).

## Discussion

Both models suggest that the contextual factors surrounding a district’s MDA campaign play a consequential role in MDA coverage. For these studied factors, so often discussed qualitatively, the model offers the ability to estimate the magnitude of such effects on coverage and compare with that of others in the process of MDA planning.

Amongst environmental factors, the effect of above average rainfall for an MDA’s time and place was small in magnitude; .27 percentage points of coverage for each additional mm of average daily precipitation. Applying this rate to the rainfall observed in this study, the net effect on coverage was less than +/- three percentage points of coverage for >99% of the 3,880 MDAs analyzed. While this estimate cannot be used to directly estimate the effect of a rainy season, the model controls for such seasonal events. Rainfall’s significant and negative effect on coverage may include both the rain itself (e.g., making roads less passable for drug distributors) as well as a reluctance of the public to engage with distributors in wetter conditions, especially when fixed point distributions are used in lieu of house-to-house distributions.

Above average temperature was associated with an increase in coverage. Roughly seven in 10 MDAs in this study saw temperatures that were higher than their climatological average. Similar to rainfall, the overall magnitude of this effect was limited: given the modelled estimate, 93.0% of the MDAs would have limited temperature’s effect to +/- three percentage points of coverage. This result was robust to the inclusion and exclusion of controls for month, rainfall, and round and warrants further investigations.

The study’s window of 2007–2020 saw two notable epidemiological events in West Africa: the Ebola virus outbreak of 2014 and the COVID-19 pandemic of 2020. These factors were the largest in magnitude of any factors studied, at –7.3 percentage points (p = .004) for Ebola and –4.7 percentage points (p = .004) for COVID-19, suggesting that MDA performance suffers notably when conducted during a public health crisis. The effect likely includes both a public reticence to interact with those outside the home as well as an operational burden assumed by drug distributors in the form of preventative measures. Ebola’s larger penalty relative to COVID-19’s may reflect the nature of the two outbreaks, with Ebola having a higher mortality rate and more severe disruption to public life, particularly in rural areas where COVID-19 did not fundamentally alter gatherings that were routinely held outside pre-pandemic, e.g., prayer, market, meals. In the case of COVID-19, the study included only MDAs conducted in late 2020—each case being a country’s first MDAs to resume after an initial hiatus from the pandemic’s onset. As such, the lower coverage observed may represent the initial adjustment period to COVID-19, whereas subsequent MDAs during the pandemic may show a return toward normal performance due to adaptation to and comfort with the new context. Further study is required to define this potential phenomenon.

The models also established where programmatic decision may affect coverage. There was a coverage benefit to conducting MDA in the same calendar month as the previous MDA. This result was robust even when controlling for each individual month. National LF programs select their month of implementation taking into account staff availability (e.g., certain months conflict with other MDAs or non-NTD work requiring staff attention), holiday periods (e.g., Ramadan), and climate (e.g., rainy season); these tend to be consistent year-to-year. Circumstances that push MDA outside this preferred calendar window may lead to calendar conflicts with other initiatives and may lose useful seasonal cues in the public’s mind that encourage participation. Since the reasons for a national LF program to choose a preferred implementation month were country or region specific, it would be difficult to interpret the modelled estimates for month (January, February, etc) as holding a more generalizable meaning for the ten countries as a whole. Month dummy variables were included in the model as control variables.

There was a clear gain in MDA coverage with successive rounds of MDA. This may reflect organizers and distributors gaining expertise through experience (e.g. better drug distributor mobilization, more timely drug donation approvals, more effective social mobilization) and/or the public becoming acclimatized to the MDA participation. All rounds (round 2, 3, 4, etc) in the model proved higher than the first round and were statistically significant in all cases (Fig 2).

There was no significant effect on MDA performance when the typically annual cycle of LF MDA was put on pause for a year or more (e.g., for reasons of insecurity, labor strikes, or for disease specific assessments that indicated more MDA is needed), suggesting that any gains attributed to experience were not lost during the hiatus.

Violence and social unrest were associated with lower coverage by three percentage points in Model 1. Specifically, this included MDAs where at least one event (event categories: battles, protests, explosions/remote violence, riots, violence against civilians, and looting/property destruction) occurred in the month of MDA or the month prior, the latter being typically when the pre-MDA social mobilization campaigns take place to alert the public of the upcoming MDA. Model 2 unpacks the unrest variable to its constituent event types. Explosions and remote violence were the only significant event types in the results, although battles, protests, riots, and violence against civilians were all similarly negative though not significant, likely due to their rarity in the dataset.

Observance of the holy month of Ramadan during or close to the MDA was not a significant factor in influencing MDA coverage. As the exact start dates of MDAs were not available for historical MDAs, this variable was specified to note potential overlap—where the month of MDA coincided with that/those of Ramadan for Muslim-majority areas. Further research may be warranted as more specific calendar data is available or the geographic areas observing Ramadan can be more granularly defined.

Two related factors, population density and isolation/road access, were examined in separate models to avoid confounding. Population density had low explanatory power toward coverage. Only Group Two (25–54 population/km2) was significantly different from the most rural group, confirming the difficulty in implementing in the most sparsely populated areas. One possible explanation for the lack of significance in the more urban groups is that MDA organizers explicitly recognize urban environments as unique challenges, adjusting MDA distribution platform and adding resources to more urban MDAs, which may obscure the underlying challenge associated with that context. More data on platform and resource allocation may elucidate a more defined relationship between population density and coverage. Isolation, significant at the 90% level, had a coefficient of -.0003 (p = .0077), which may indicate that a district with road access for all of its population is expected to achieve coverage 3 percentage points higher than a district entirely without road access, all other things being equal. This aligns with the intuitive notion that the remoteness of communities poses a challenge for implementers. Since community drug distributors are often recruited to cover their own or nearby communities, this finding may indicate a supply chain challenge for drug inventory.

Findings are caveated by known limitations in the underlying datasets. The coverage data, sourced from the USAID NTD database, reflects unaltered population estimates by national LF programs. As programs switch from one source of population data to another, variation is introduced to coverage that is not related to MDA performance, obscuring the effects of contextual factors in the model. Investigating each instance of district-level population deviation was beyond the scope of this study, and an attempt to smooth population growth removed valid variation along with that from data source switching and so was not included in the final analysis.

The data is also limited to the window of 2007–2020; where countries have experience implementing earlier LF MDAs, such dynamics are not captured in the model. Similarly, post-2020 MDAs and covariates are not captured. This is particularly relevant to the estimation of COVID-19’s role in MDA, as only the first of several COVID-concurrent MDAs were included. Inclusion of subsequent COVID-concurrent MDA data may reveal a smaller COVID penalty as MDA organizers and the public adapt to the challenges of the pandemic.

This dataset also tracks districts longitudinally by design. In cases where a district has split into two or more “daughter” districts in a given year, the dataset was updated to display coverage for the daughter districts for all years, even those before the daughters’ creation. Districts that split during this study effectively have their pre-split coverage rates counted more than once in the model, while the inverse was true for districts that merged during the years of this study. This applied to less than 10% of the MDA results in this study. While this adjustment deviates the dataset from more authoritative sources (e.g., WHO LF elimination dossiers), it enables closer longitudinal analysis for the purposes of this study. The dataset should therefore not be considered an official record of a country’s LF MDA history.

The ACLED data on violence is a robust source of information, yet some acts of violence may likely go unreported or be overreported depending on how reports are made. Since the number of violent events were added together for each district by month for our analysis, the results may not be fully accurate.

Overall, the results of both models broadly aligned with intuition when characterizing the type of effect, positive or negative, of specific contextual conditions on MDA performance. The novelty in this analysis lies in the model’s ability to quantify a magnitude for these effects on coverage. This quantification allows different factors to be compared directly (e.g., pausing MDA for a year’s hiatus vs potential overlap with Ramadan). Of the factors studied, the greatest challenges to coverage, by magnitude, were Ebola, COVID-19, and violence/unrest. These were associated with seven, five, and three percentage points drops in coverage, all else being equal. The most beneficial precondition for a higher coverage MDA was experience, i.e., the number of rounds previously conducted in a given district. This benefit was strongest between six and twelve rounds which were roughly nine percentage points higher than the first round’s coverage.

By quantifying these associations, the findings may assist MDA organizers to better prioritize certain factors when seeking out ideal circumstances for future MDA. Notably the non-significant cost of a hiatus from annual treatment, compared to the rather steep and significant penalties associated with unrest or public health emergencies may move MDA organizers to exercise caution where disruptive events are likely. Such decisions must be made in concert with MDA epidemiological models, however, as more time between rounds of MDA has implications for disease transmission.

Additional research is needed to explain the apparent positive effect of higher-than-normal temperatures on coverage which, paired with precipitation findings, should offer insight on the effects of climate change on future MDAs. Further investigation is also warranted on MDA delivery platforms (e.g., fixed post vs door-to-door distributions) and CDDs themselves (e.g., CDD-to-population ratio, training types, demographic profiles) to enhance the utility of these findings for MDA planning.

As an approach, the coverage model was successful in assigning quantitative estimates to typically anecdotal explanations for variation in coverage. The hierarchical linear model, or other models like it, should be considered a tool alongside coverage evaluation surveys in understanding the reasons behind high and low coverage. Methodologically, this approach can be applied to regions outside West Africa and to factors outside those included above to better understand MDA coverage, particularly for diseases that depend on successful MDA for their elimination as a public health problem.
